# Aggressive Angiomyxoma of the Vulva: A Diagnostic Dilemma

**DOI:** 10.7759/cureus.111531

**Published:** 2026-06-26

**Authors:** Pruthwiraj Sethi, Chandra Jyoti, Pritinanda Mishra

**Affiliations:** 1 Obstetrics and Gynecology, All India Institute of Medical Sciences Bhubaneswar, Bhubaneswar, IND; 2 Pathology, All India Institute of Medical Sciences Bhubaneswar, Bhubaneswar, IND

**Keywords:** aggressive angiomyxoma, angiomyxoma, local neoplasm recurrence, mesenchymal tumors, vulval tumor

## Abstract

Aggressive angiomyxoma is a rare mesenchymal tumor involving soft tissue of the vulvovaginal and perineal region in females of reproductive age. A 42-year-old, para-2 female presented with an elongated swelling on the left labia majora for 10 years. She presented to us because she was feeling uncomfortable while sitting. Local examination showed an elongated mass measuring 8 x 6 cm in the longitudinal direction protruding from the lower part of the left labia majora. The mass was soft, spongy, and non-tender without any involvement of adjacent structures. Sonography of the local area showed an echogenic soft-tissue mass with increased color flow, likely a lipoma of the vulva. She underwent a wide local excision of the tumor. On histopathology, it was diagnosed as an aggressive angiomyxoma of the vulva.

Angiomyxoma arises from the mesenchymal tissue, and it is locally invasive with a high recurrence rate. It has to be differentiated from Bartholin cyst, lipoma, Gartner cyst, and pelvic hernia. The diagnosis can be aided by imaging modalities such as ultrasonography, magnetic resonance imaging, and computed tomography. Wide excision is the treatment of choice. Histology, along with immunohistochemistry, can confirm the lesion. Long-term follow-up is needed as recurrences are high.

## Introduction

Aggressive angiomyxoma is a rare mesenchymal tumor involving soft tissue of the vulvovaginal and perineal region in females of reproductive age [1]. In 1983, Steeper and Rosai first described this entity and reported a case series of nine patients [2]. Angiomyxoma presents as a slow-growing tumor and mostly has a delayed presentation. Wide local surgical excision of the mass followed by histopathological examination remains the cornerstone for the diagnosis and management of aggressive angiomyxoma. As per the WHO classification, aggressive angiomyxoma is classified as a "Tumor of Uncertain Differentiation"[3]. The term 'aggressive' refers to its locally infiltrative nature and tendency for recurrence. It has a higher risk of local recurrence, which may vary from 30% to 72% [4]. Due to its rarity, there is no definite consensus on the clinical presentation and treatment protocol in the recent literature. We report a case of vulvar aggressive angiomyxoma diagnosed following surgical excision.

## Case presentation

**Figure 1:**
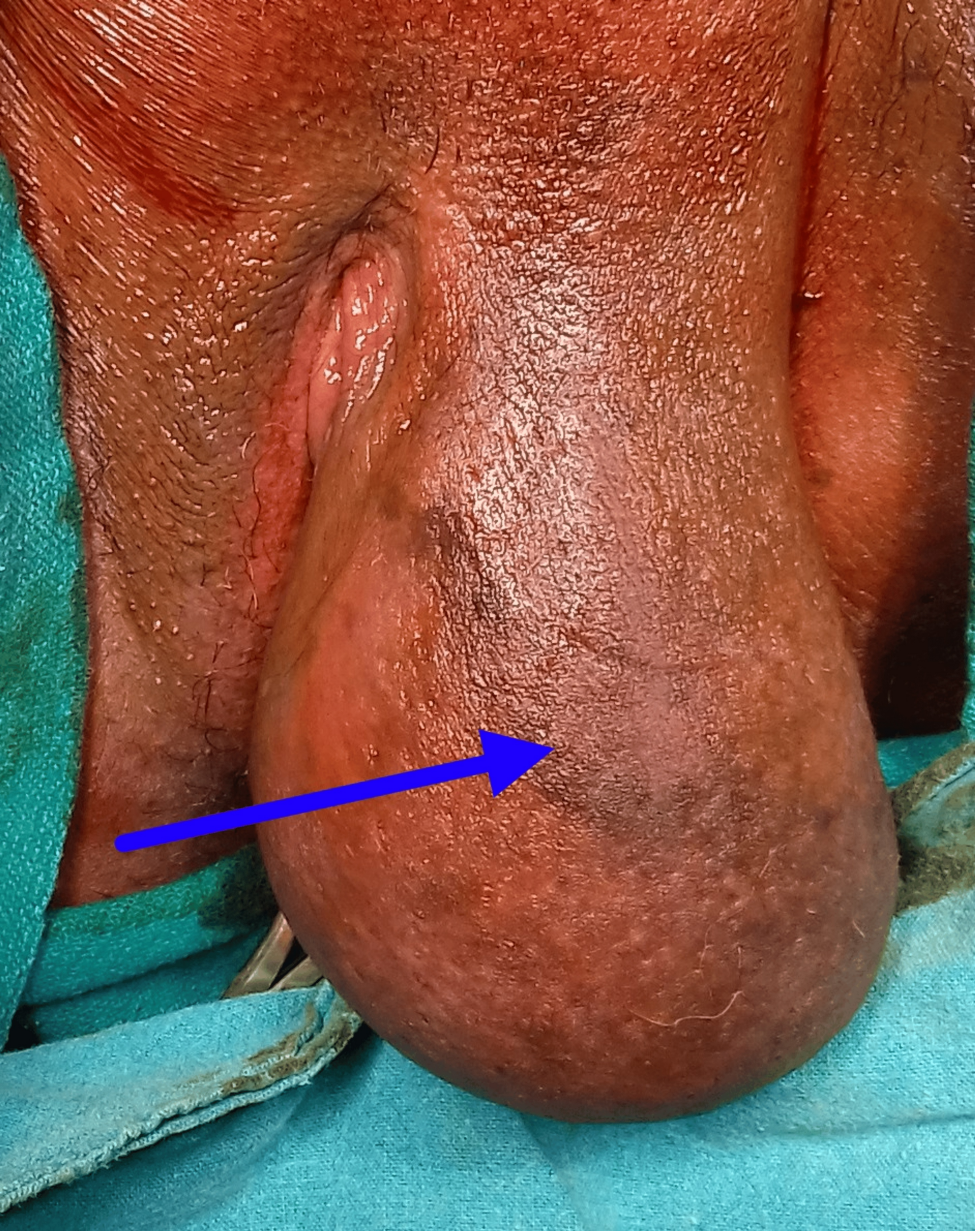
Mass arising from the left labia majora (the blue arrow indicates the mass)

A 42-year-old, para-2 female presented with an elongated swelling on the left labia majora for 10 years. It started with a small swelling with slow progression. She presented to us because she was feeling uncomfortable while sitting. Her past medical and surgical histories were unremarkable. Local examination showed an elongated mass measuring 8 x 6 cm in longitudinal direction, protruding from the lower part of the left labia majora (Figure 1).

**Figure 2:**
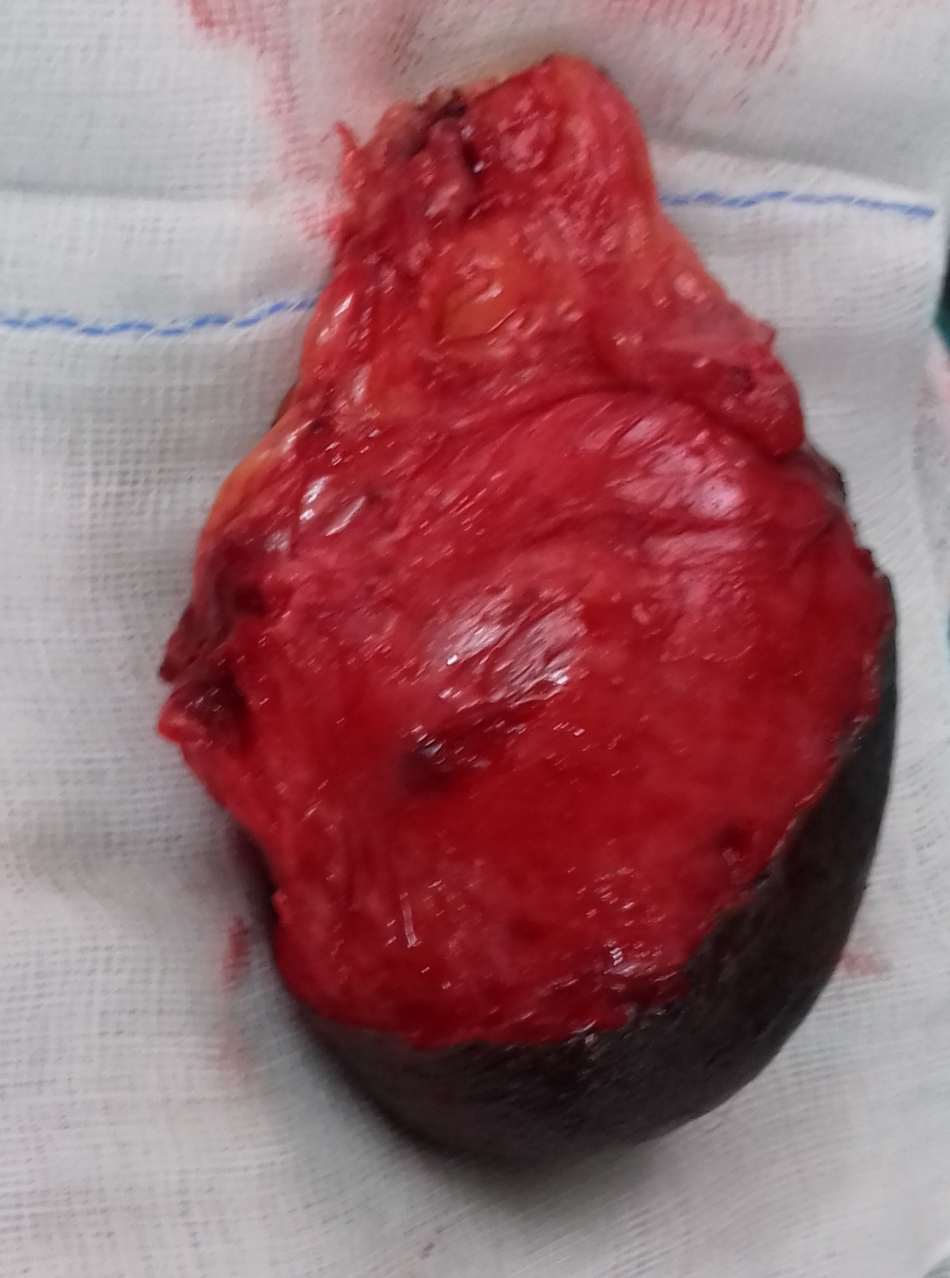
Gross specimen of angiomyxoma, measuring 8 × 6 × 5 cm and consisting of a poorly circumscribed, unencapsulated soft tissue mass

The mass was soft, spongy, and non-tender with intact overlying skin integrity. Sonography of the local area showed an echogenic soft-tissue mass with increased color flow, likely to be a lipoma of the vulva. She underwent a wide local excision with a gross surgical margin of 1 cm from the surrounding healthy tissue. Intraoperatively, complete delineation of the tumor was challenging at its cephalic aspect because of indistinct tissue planes and extension into the surrounding soft tissues. Grossly, the specimen measured 8 × 6 × 5 cm and consisted of a poorly circumscribed, unencapsulated soft tissue mass (Figure 2).

**Figure 3:**
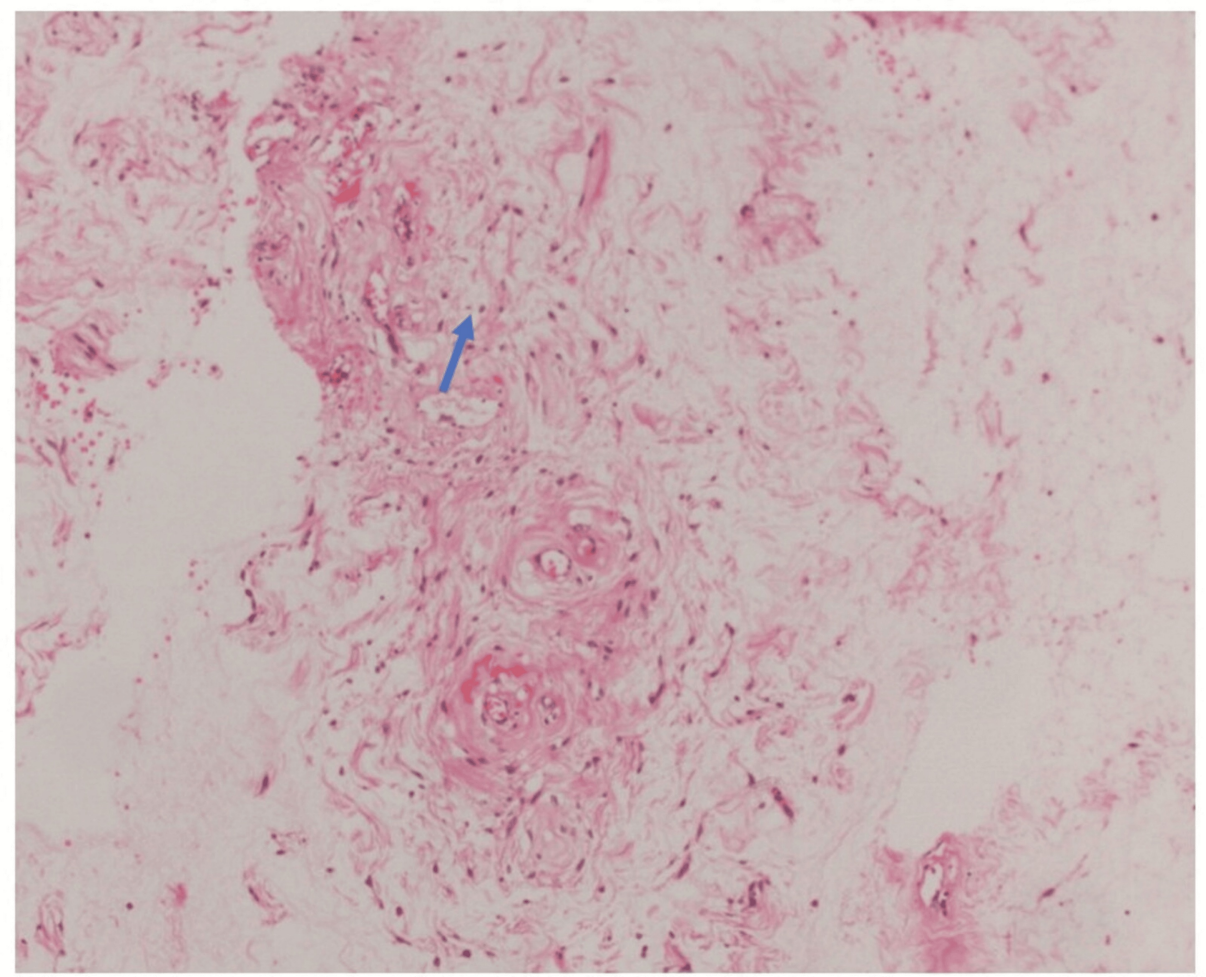
Photomicrograph showing hypocellular spindled (blue arrow) and stellate fibroblasts with no atypia Hematoxylin and Eosin stain, ×100; scale bar = 100 μm

**Figure 4:**
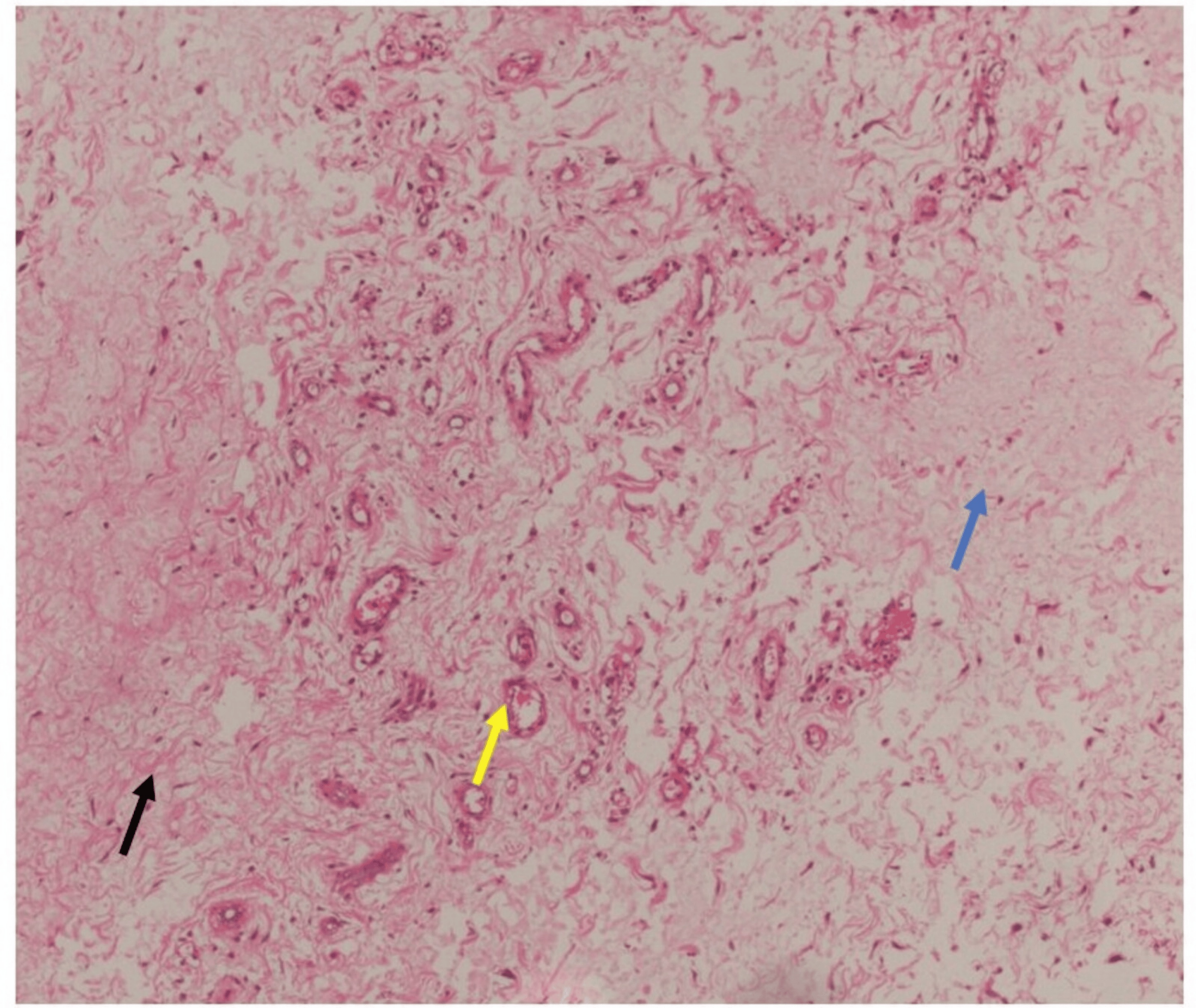
Photomicrograph showing myxoid stroma (blue arrow) with collagen fibers (black arrow) and prominent, dilated, thick-walled vessels (yellow arrow) Hematoxylin and Eosin stain, ×100; scale bar = 100 μm

On histopathology, the tumor was paucicellular, composed of oval-to-spindle-shaped cells with bland nuclei and scant cytoplasm in a myxoid stroma with numerous blood vessels of variable calibre. No cellular atypia was noted, and the base was free from tumor (Figure 3, Figure 4).

Immunohistochemistry was not performed because it was not available at that time. Considering the typical histopathological features, it was diagnosed as an aggressive angiomyxoma of the vulva. She was discharged from the hospital in good condition, with advice to attend six-monthly follow-ups. No recurrence was observed after two years of follow-up since the last surgery.

## Discussion

Aggressive angiomyxoma (AAM) is a slow-growing soft-tissue tumor with a potentially locally infiltrating nature, usually found in the pelvis and perineal regions of women of reproductive age. They present as soft, non-tender, rubbery, and are frequently confused with Bartholin cysts, Gartner duct cysts, lipomas, or perineal hernias [5]. This tumor is slow-growing, and the patient seeks help when there is pain or ulceration. This diagnostic challenge often leads to delayed recognition and inappropriate initial management. In our case, a preoperative diagnosis of lipoma was made from clinical examination and ultrasonography findings.

Unlike ultrasonography or computed tomography (CT), magnetic resonance imaging (MRI) is superior in delineating the infiltrative margins of the lesion and its relation to adjacent pelvic and perineal structures. This information is vital for determining the possibility of achieving complete excision. On MRI, these tumors show high signal intensity on T2-weighted images with a characteristic 'swirled' pattern due to high content of loose myxoid matrix [6]. In our case, CT or MRI was not performed prior to surgery, as we were thinking of a lipoma. This can be considered as a limitation of the case, as the full extent of the disease could not be determined before surgical excision.

On gross cut section, the tumors are tan-gray to pink, gelatinous, glistening, with a rubbery consistency [7]. In histopathology, the tumor is paucicellular, composed of oval-to-spindle cells with bland nuclei and scant cytoplasm, in a myxoid stroma with numerous blood vessels of variable caliber [5]. There is no cellular atypia, with low mitotic activity. Ki-67 indices in aggressive angiomyxoma are generally low, due to the tumor's indolent growth pattern [8]. Immunohistochemistry of tumor cells shows expression of desmin, smooth muscle actin, vimentin, and CD34 [9]. They are also positive for estrogen and progesterone receptors. In the present case, immunohistochemistry and Ki-67 indices were not evaluated due to unavailability, which is a limitation of this case report. However, the diagnosis was established predominantly based on the characteristic morphology and clinicopathological correlation. The differential considered was angiomyofibroblastomas, which show a well-circumscribed margin and are composed of alternating hypercellular and hypocellular areas [10]. The other histological differential diagnosis includes myxoma, myxoid liposarcoma, myxofibrosarcoma, and nerve sheath myxoma. Marked vascularity and typical infiltrative nature in aggressive angiomyxoma help differentiate it from most of the above neoplasms [11].

The primary treatment for aggressive angiomyxoma is surgical excision with tumor-free margins. Due to the infiltrative growth pattern of the tumor and its proximity to nearby pelvic structures, achieving wide margins may be difficult and can result in significant morbidity [11]. However, recurrence may occur even after excision of the tumor with negative margins [12].

The role of hormonal therapy in the management is considered due to receptor positivity, particularly estrogen and progesterone receptors, in aggressive angiomyxoma. Treatment with gonadotropin-releasing hormone (GnRH) analogs and aromatase inhibitors has been used in patients with recurrent, residual, or unresectable tumors, which helps in tumor regression or prevents recurrence [13,14]. In postmenopausal women, aromatase inhibitors may be particularly relevant because they suppress peripheral estrogen production [15,16].

Other treatment modalities include embolization of the mass and radiotherapy. Embolization of the mass may be used in large tumors to reduce their size, allowing surgery with less morbidity. Radiation therapy has been used in cases of recurrence or to reduce the recurrence postoperatively.

In recent literature, recurrence rates vary from 30% to 72%, with most recurrences occurring within 3 years, but it has been described at up to 14 years [4]. Therefore, it is recommended to have an extended follow-up, both clinical and radiological, beyond three to five years.

## Conclusions

Angiomyxoma is a rare, slow-growing, and locally infiltrative tumor predominantly found in the perineal region in women of reproductive age. Wide local excision of the tumor is the primary modality of treatment. Due to the high risk of local recurrence, adjuvant hormone therapy should be considered in these patients. Awareness of this entity among gynecologists, radiologists, and oncologists is crucial for proper diagnosis, management, and long-term follow-up.

## References

[1] Amezcua CA, Begley SJ, Mata N, Felix JC, Ballard CA (2005). Aggressive angiomyxoma of the female genital tract: a clinicopathologic and immunohistochemical study of 12 cases. Int J Gynecol Cancer.

[2] Steeper TA, Rosai J (1983). Aggressive angiomyxoma of the female pelvis and perineum. Report of nine cases of a distinctive type of gynecologic soft-tissue neoplasm. Am J Surg Pathol.

[3] Bai HM, Yang JX, Huang HF (2013). Individualized managing strategies of aggressive angiomyxoma of female genital tract and pelvis. Eur J Surg Oncol.

[4] Qu H, Liu N, Liang H, Wang Y, Zhuang H, Li H (2024). Aggressive angiomyxoma of female pelvis and perineum: retrospective study of 17 cases. Eur J Obstet Gynecol Reprod Biol.

[5] Salman MC, Kuzey GM, Dogan NU, Yuce K (2009). Aggressive angiomyxoma of vulva recurring 8 years after initial diagnosis. Arch Gynecol Obstet.

[6] Surabhi VR, Garg N, Frumovitz M, Bhosale P, Prasad SR, Meis JM (2014). Aggressive angiomyxomas: a comprehensive imaging review with clinical and histopathologic correlation. AJR Am J Roentgenol.

[7] Narang S, Kohli S, Kumar V, Chandoke R (2014). Aggressive angiomyxoma with perineal herniation. J Clin Imaging Sci.

[8] Chen H, Zhao H, Xie Y, Jin M (2017). Clinicopathological features and differential diagnosis of aggressive angiomyxoma of the female pelvis. 5 case reports and literature review. Medicine (Baltimore).

[9] McCluggage WG (2005). A review and update of morphologically bland vulvovaginal mesenchymal lesions. Int J Gynecol Pathol.

[10] Nucci MR, Fletcher CD (2000). Vulvovaginal soft tissue tumours: update and review. Histopathology.

[11] Goyal LD, Garg P, Badyal R, Bhalla S (2022). Aggressive (deep) angiomyxoma of the vulva: a case report. J Med Case Rep.

[12] Sengupta SK, Bhattacharyya SK, Saha SP, Roy H, Sarkar AN (2014). Recurrent aggressive angiomyxoma of the vulva - a rare presentation. J Clin Diagn Res.

[13] Saito A, Komiyama S, Nagashima M, Kugimiya T, Mukai T (2025). Recurrent aggressive angiomyxoma that responded to the gonadotropin-releasing hormone (GnRH) antagonist relugolix. Cureus.

[14] Gorgulu G, Kole MÇ, Ayaz D, Kuru O, Gokcu M, Sanci M (2022). Aggressive angiomyxoma. A case series of eight years of experience. Ann Ital Chir.

[15] Maciejczyk A, Bartecki K, Czarnecka AM, Szumera-Ciećkiewicz A, Rutowski P, Świtaj T (2025). Hormonal treatment of aggressive angiomyxoma. Curr Probl Cancer.

[16] Yu BR, Choi WK, Cho DH, Lee NR (2025). Aggressive angiomyxoma of the vagina: a case report and literature review. Medicine (Baltimore).

